# Effect of Thermal Ageing on the Impact and Flexural Damage Behaviour of Carbon Fibre-Reinforced Epoxy Laminates

**DOI:** 10.3390/polym11010080

**Published:** 2019-01-07

**Authors:** Irene García-Moreno, Miguel Ángel Caminero, Gloria Patricia Rodríguez, Juan José López-Cela

**Affiliations:** Escuela Técnica Superior de Ingenieros Industriales, INEI, Universidad de Castilla-La Mancha, Campus Universitario s/n, 13071-Ciudad Real, Spain; irene.gmorero@uclm.es (I.G.-M.); gloria.rodriguez@uclm.es (G.P.R.); juanjose.lopez@uclm.es (J.J.L.-C.)

**Keywords:** thermal ageing, thermoset matrix, carbon fibre-reinforced composites, glass transition temperature, Charpy impact response, flexural response

## Abstract

Most of the composite materials that are used in aerospace structures have been manufactured using a thermostable matrix, as epoxy resin. The region of stability of these polymers is defined by the glass transition temperature (*T*_g_). However, operating temperatures close and above the *T*_g_ can cause a variation in the properties of the polymer and consequently, modify the mechanical properties of the composite material. Therefore, it is necessary to understand the failure mechanisms that occur in the material in order to ensure stability and durability. The effect of temperature and time of exposure on the impact and flexural mechanical responses of carbon/epoxy composites are studied in this work. For that purpose, ageing treatments at temperatures below and above the *T*_g_ have been considered and then, impact and flexural tests have been performed. It was observed that thermal ageing cause two different effects: at temperatures below the *T*_g_, there is an increase of the maximum strength because of a post-curing effect; however, the mechanical properties decrease at higher temperatures of thermal ageing due to the thermo-oxidation of the epoxy resin and the loss of adhesion in the matrix/fibre interface.

## 1. Introduction

Fibre-reinforced composite materials have become relevant in aerospace, automotive, wind energy, marine and civil engineering applications due to their high specific strength, stiffness and fatigue performance [1,2,3,4,5,6]. However, a major concern of carbon fibre-reinforced polymer (CFRP) structures is how environmental conditions affect the material properties and consequently the in-service behaviour and durability of the structure. Composite structures present an inherent sensitivity to environmental factors compared to metallic structures, so they must be designed considering the influence of factors such as moisture, temperature or UV radiation to ensure the reliability and safety of CFRP structures [7].

In many applications, such as aeronautical components, one of the greatest concerns is associated with the long-term ageing effect, since oxidation strongly affects the properties of the polymer matrix, particularity failure performance. Fibres are relatively stable but the matrix and the interface can degrade with temperature. Thermal ageing of reinforced thermoset matrix composites is frequently associated with a physicochemical degradation of the resin that involves several changes on the thermomechanical properties of composites [8]. Glass transition temperature (*T*_g_) of a thermoset polymer defines the region where the material present high stability in mechanical and thermal properties. Although the short-term mechanism of ageing of CFRP are known, in the long term there are many gaps regarding these mechanisms that represent an obstacle to the development of these materials [9].

The ageing process of polymers have been classified into two categories: physical and chemical ageing. Physical ageing is a reversible process that occurs during prolonged exposures of the material at elevated temperatures below the glass transition temperatures (*T*_g_) and implies changes in the molecular conformation of the material without changing the structural integrity of the molecules. On the contrary, chemical ageing of the resin is an irreversible degradation that leads to a reduction in the molecular weight because of changes in the cross-link density, oxidation and depolymerisation [7,10,11,12,13].

Many studies regarding thermal ageing of carbon-epoxy composites are available in the literature [14,15,16,17,18,19,20,21,22,23,24,25]; however, the effect of ageing and how the mechanical properties of the aged materials are modified are not completely understood [20]. According to the available literature, a slight increase in the mechanical properties of CFRP is observed at the first stage of ageing that is called consolidation phase. Such improvement is attributed to different post-curing reactions that are favoured by the increase in temperature [7]. The high temperature exposure reactivates the molecular mobility in the polymeric matrix what implies an increase of the cross-link density in the material structure and consequently, the glass transition temperature increases during the consolidation phase [9]. After this initial stage, a degradation phase starts and the mechanical properties of the composites significantly decrease due to matrix deterioration and weakening of the fibre-matrix interface. Cracks appear and propagate rapidly, causing a reduction of the *T*_g_ [7,9]. Oxidation occurs principally on de surface of the composite along fibre direction and mismatches in the coefficients of thermal expansion between the fibres and matrix during the composite cure process, cause the fibre/matrix debonding and create new paths for oxygen to penetrate during thermal ageing [26]. Oxidation can cause the embrittlement of epoxy resins, which produce the growth of microcracks at lower applied loads and strongly affect the epoxy mechanical properties [27]. Failure properties are governed by the capacity to promote plastic deformation. Although plastic deformation in glassy state have been studied for various decades, the molecular origin for plastic deformation mechanisms in epoxy resin is not completely understood [8]. The effect of thermal ageing on the elastic modulus is not clear, some researches have not observe any effect while others have noticed modest variations of the elastic modulus with ageing [28,29,30].

In addition, composite structures remain vulnerable to low-velocity impact damage, so several studies have published in the literature focused on this topic [31,32,33]. However, it could be interesting to study the influence of thermal ageing on the low-velocity impact response of CFRP structures. These types of composite systems have service temperatures around 100–120 °C on continuous basis and around 135 °C for short duration. For most structural applications in the current aircraft designs, this has been adequate and has led to successful applications. However, there are several frame structural components in an aircraft, such as aero-engine covers and leading edge flaps, which can be exposed to higher temperatures than the usual service temperatures. Hence, it is of great concern to evaluate the degradation of the mechanical performance (impact and flexural behaviour) of this type of polymer-based composite systems when they exceed the usual operating temperature values. Further, high-speed transport aircraft structures in future, particularly leading edges, may be required to endure quite high temperatures (350 °C or above) as higher speeds and space re-entry features will become a part of the aeroplane designs. As the polymer matrix material is the most affected (rather than the reinforcing fibres such as glass or carbon) by high temperature, the matrix material will be the focus of attention in the development of high-temperature polymer-matrix composites. Despite de wide variety of works published on literature [34,35,36,37], the results are ambiguous and inconclusive. This situation can be explained by the absence of standardized methods and the use of different fibre/matrix combinations and different ageing treatments leading to inconsistent results [9]. Furthermore, flexural behaviour is normally used to evaluate the properties of composite laminates because they are subjected to both compressive and tensile stress. Due to fibre misalignment and manufacturing defects, the compressive modulus of long fibre composites is reasonably not expected to be equal to the tensile modulus. The effects of unequal compressive/tensile response of the failure performance of composites have already been studied [38,39] but the effect of thermal ageing on the flexural strength and stiffness has been studied in a very limited number of works.

In this paper, the effect of thermal ageing at different temperatures and time of exposure on the impact and flexural properties of carbon/epoxy composites is studied. The glass transition temperature of the epoxy resin considered in this study corresponds to 195 °C. Temperatures below and above this value were considered. Instrumented Charpy tests were performed in order to determine the impact behaviour of aged specimens and three-point bending tests were used to evaluate the flexural properties. Optical micrographs of the cross-sections of the failed samples were examined to gain an insight into the assessment of different failure mechanisms.

## 2. Materials and Methods 

### 2.1. Materials and Specimen Preparation

The specimens were manufactured from commercially available carbon/epoxy pre-impregnated tapes (Hexcel Composites Ltd., Stamford, CT, USA). The prepreg tapes were made of unidirectional (UD) continuous high tensile strength carbon fibres IMA-12K, pre-impregnated with Hexply^®^ M21E thermosetting epoxy, a high performance, very tough epoxy resin used in the primary structure of the Airbus A350 XWB, because of the excellent damage tolerance. It has a resin content of 34% by weight and a fibre weight of 268 g/m². The basic in-plane stiffness and strength of the M21E/IMA unidirectional laminate under tensile and compressive loading are presented in Table 1 [31,38]. The final properties of the composites depend on the manufacturing process. In this study, the material was laid up by hand with different stacking sequences: cross ply [0/90]_4s_ and quasi-isotropic [0/90/(±45)]_2s_ laminates with a total of 16 plies and 4 mm of thickness. 

The laminates were cured following the standard cure cycle recommended by Hexcel Composites Ltd. [40] at 7 bar hot-pressing system and 180 °C of temperature. The glass transition temperature for the epoxy resin provided by the materials manufacturer Hexcel Composite Ltd. is 195 °C and it has a service temperature up to 150 °C. The composite plates were ultrasonically C-scanned to verify the quality and structural integrity.

**Figure 1:**
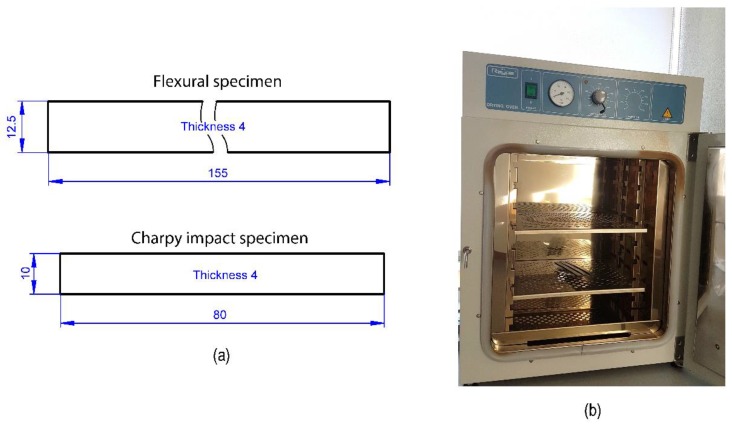
(**a**) Standard flexural and Charpy impact specimens according to ASTM D7264 and D6110 or ISO 179 recommendations. (**b**) Programmable oven used for the thermal ageing treatments.

Two types of samples were cut from the composite plates: 80 × 10 × 4 mm samples for Charpy samples and 155 × 12.5 × 4 mm samples for three-point bending samples (Figure 1a). The dimensions of Charpy impact and three-point bending samples were defined following the dimensions of previous works [38] and the recommendations of the standards ASTM D6110 [41] or ISO 179 [42] for Charpy test and ASTM D7264 [43] for three-point bending test. Quasi-isotropic specimens have been subjected to thermal ageing in a drying oven (Figure 1b) at different temperatures above and below the glass transition temperature as it is described in Table 2. Different exposure time and temperature values were considered in the thermal ageing treatments for Charpy samples in order to study the influence of both effects. However, in the case of flexural samples, thermal ageing treatments with different temperatures were considered but with a fixed exposure time of 10 days. Charpy impact and flexural tests were performed according to the ASTM D6110 or ISO 179 and D7264 methods respectively. For each sample, three specimens were tested and the average values were taken as the result.

### 2.2. Low Velocity Impact Testing

**Figure 2:**
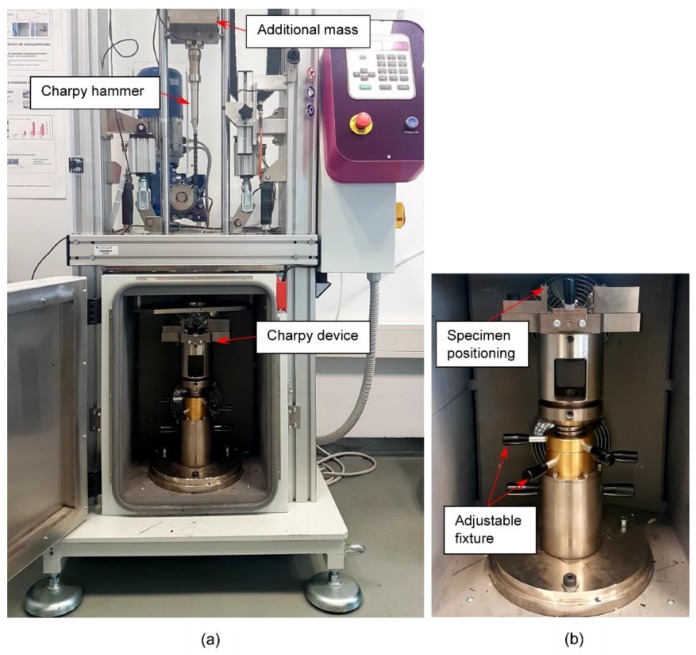
Experimental set-up for drop-weight impact test. (**a**) Drop-weight column device and (**b**) Charpy support fixture according to ASTM D6110.

**Figure 3:**
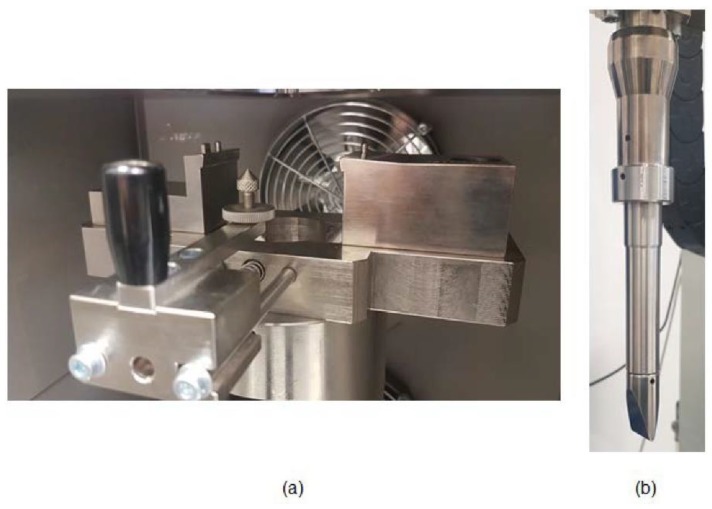
Details of the Charpy testing accessories according to ASTM D6110 used in the drop-weight column. (**a**) Charpy device support and (**b**) Charpy impactor (20 mm × 4 mm).

Instrumented Charpy impact test were performed in a drop weight column CEAST 9340 (Instron Ltd., Norwood, MA, USA) using Charpy testing accessories following ASTM D6110, ISO 179 and ASTM E23 standards. These Charpy fixtures (Charpy impactor and Charpy support device) are depicted in Figure 2 and Figure 3. It is clearly stated in the previous standards that the method should not be used to obtain design data. The purpose of the Charpy test in this work was to provide a comparative test to evaluate the local impact absorption due to different thermal aging treatments. Impact conditions can be defined as a function of the mass, velocity or drop height. The maximum drop mass available is 37.5 kg and the energy range is from 0.88 to 405 J. All the impact tests in this work followed the impact conditions detailed in Table 3. The impactor was dropped from a selected height and impacted at the centre of the specimen causing the total break. The values of load, energy, time and displacement were recorded during every test with the CEAST DAS 64k acquisition system (Instron Ltd, Norwood, MA, USA), thus the damage resistance can be evaluated using the recorded impact histories of force-time, force-displacement and energy-time. Maximum force and maximum energy can be obtained from the curves in order to compare the impact response of different ageing treatments.

### 2.3. Three-Point Bending Tests

**Figure 4:**
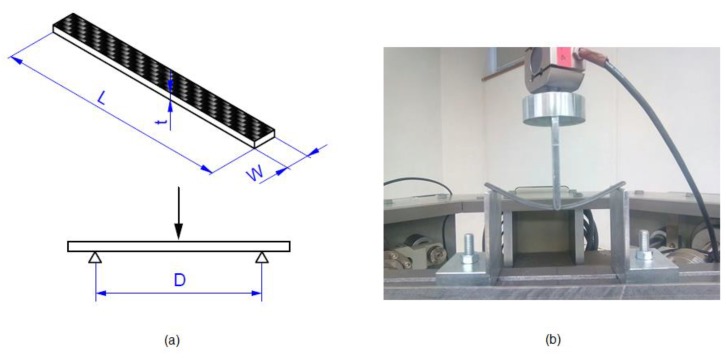
Details of the 3-point bending test. (**a**) Span sample dimension (*D* = 130 mm) and (**b**) experimental setup according to ASTM D7264.

Three-point bending tests were performed using a 50 kN universal electro-mechanical testing machine with a 5 kN load cell at a fixed loading rate of 2 mm/min (Figure 4), according to the ASTM D7264. The radius of the loading nose and the radii of the support noses of the 3-point bending specimen test fixture were 3 mm. The 3-point bending specimen preparation and testing is relatively simple but the results are sensitive to the geometry of the sample. Both the normal stress and shear stress in bending depend strongly on the *D/t* ratio, where *D* = 130 mm is the span and *t* = 4 mm is the thickness. Either bending or shear failure modes can appear depending on the *D/t* ratio. The standard ASTM D7264 recommends a D/t ratio greater than 32 in order to guarantee flexural failure. This limit has been considered in the dimensions of the flexural specimens. The flexural stress *σ*, flexural strain ε and flexural modulus *E*, were calculated using the following expressions, according to the standard ASTM D7264. This standard is based on the Classical Beam Theory (CBT), supposing that shear effects are negligible,

(1) $$\sigma =\frac{3PD}{2bh^{2}}$$

(2) $$\varepsilon =\frac{6\delta h}{D^{2}}$$

(3) $$E=\frac{D^{3}m}{4bh^{3}}$$

where *P* is the load at a given point on the load-deflection curve; *D* is the span, *b* is the width of the sample tested; *h* is the thickness of the sample, *δ* is the maximum deflection of the centre of the sample and *m* is the slope of the tangent to the initial straight-line portion of the load-deflection curve. In this work, the purpose of the three-point bending tests was to determine the flexural performance of CFRP samples after being aged at different temperatures.

## 3. Results and Discussion

The main effects of thermal ageing on the impact damage resistance and flexural behaviour are summarized in the following sections. 

### 3.1. Effect of Thermal Ageing on the Impact Damage Performance

Charpy impact tests were performed in a drop weight column in order to study the influence of thermal ageing on the impact damage response of CFRP laminates. The effect of temperature and time of ageing are particularly studied in this section.

**Figure 5:**
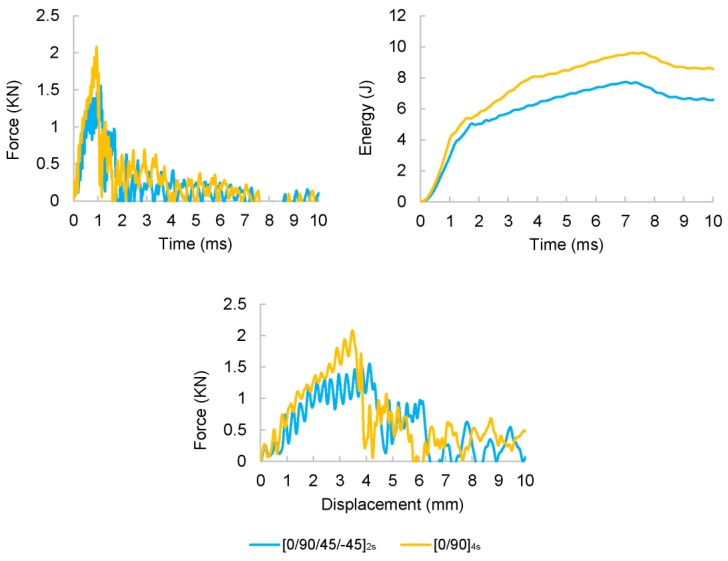
Average force and energy histories and force-displacement curves of non-thermal aged [0/90/±45]_2s_ laminates (*E*_impact_ = 32.46 J).

Firstly, some initial tests were performed in non-aged laminates with the aim of determining the impact behaviour of CFRP specimens and using the corresponding absorbed energy values as reference. For that purpose, the study of the impact response was performed in quasi-isotropic [0/90/±45]_2s_ and cross ply [0/90]_4s_ laminates. Some representative force-time, energy-time histories and force-displacement curves are depicted in Figure 5. Table 4 shows the maximum force and maximum absorbed energy per unit cross-sectional area (or impact strength *E*_c_) results of the laminates subjected to an impact energy of 32.46 J. 

**Figure 6:**
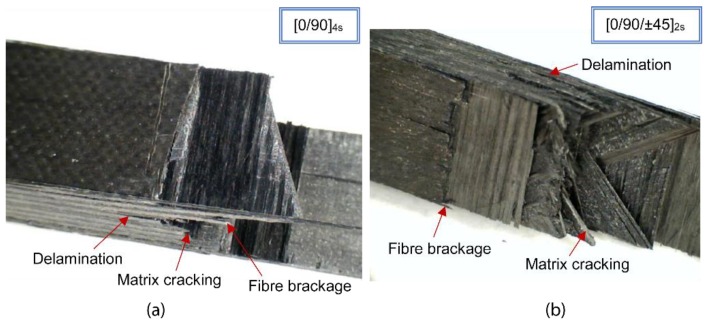
Failure modes observed in non-aged (**a**) [0/90]_4s_ and (**b**) [0/90/±45]_2s_ laminates (*E*_impact_ = 32.46 J).

The results highlighted that the averaged impact strength of cross-ply specimens (23 ± 6 J/cm^2^) was slightly higher than the corresponding to quasi-isotropic specimens (20 ± 1 J/cm^2^) but with a greater dispersion. However, there were not significant differences in the failure modes observed for the two configurations. In both cases, fibre breakage as well as matrix cracking and delaminations were observed around the impact area (Figure 6). The influence of the stacking sequence on the absorbed energy and impact strength was analysed in previous works [44] and it was observed that multidirectional specimens depicted higher values of absorbed energy and the failure modes were a combination of those showed in unidirectional ones. The presence of 0° fibre layers provided enhanced impact behaviour and highest values of absorbed energy. Although quasi-isotropic [0/90/±45]_2s_ laminates showed pseudo-ductile behaviour [38,44,45] due to the shearing effect of the 45°, the number of 0° layer was smaller compared to cross ply [0/90]_4s_. These results were in agreement with previous studies in which cross-ply laminates depicted the highest energy absorption and exhibited the maximum impact resistance compared to unidirectional and quasi-isotropic composite laminates [46]. Although cross-ply laminates depicted an improved impact performance, it was decided to study the thermal ageing effect on quasi-isotropic laminates in order to reduce the dispersion and identify with more precision the effect of thermal degradation. 

**Figure 7:**
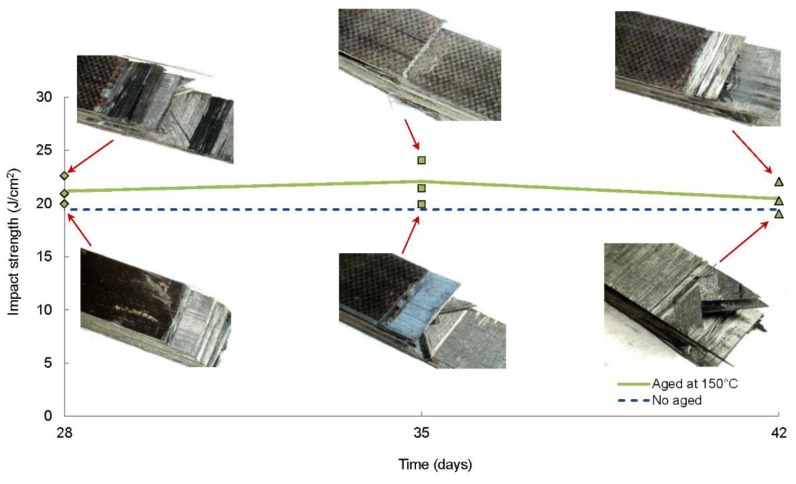
Average impact strength (E_c_) and failure modes of quasi-isotropic [0/90/±45]_2s_ laminates aged at 150 °C for different periods: 28, 35 and 42 days (*E*_impact_ = 32.46 J).

The results obtained from the impact tests of aged laminates at 150 °C for long periods of time are shown in Figure 7. This temperature was below the glass transition temperature (195 °C) and the carbon-epoxy composite laminate was supposed to maintain the mechanical properties, as the manufacturer indicates. For that purpose, ageing treatments at 150 °C for 28, 35 and 42 days followed by impact tests were performed on quasi-isotropic laminates. The average maximum force and impact strength for the different time of ageing were summarized in Table 5. The failure modes observed were a combination of fibre breakage and delamination (Figure 7). The average values of impact strength were plotted in order to observe a possible trend in the impact performance. No degradation was noticed in the impact performance of quasi-isotropic specimens regardless the time of exposure at 150 °C, as can be deduced from the close values of absorbed energy for the different periods of ageing. On the contrary, a slight improvement in the impact strength with respect to the non-aged specimens (19.62 J/cm^2^) occurred, particularly for the specimens aged at 150 °C for 35 days (22.06 J/cm^2^) in which the increase in impact strength was about 12.5%. The same behaviour was observed in previous studies, where they observed that before the degradation of the mechanical properties of carbon fibre-reinforced composites, a consolidation phase occurred related to a post-curing process [20,47]. The exposure to temperatures below the glass transition temperature ensured better adhesion between the matrix and the fibres and improved the impact response of this material.

**Figure 8:**
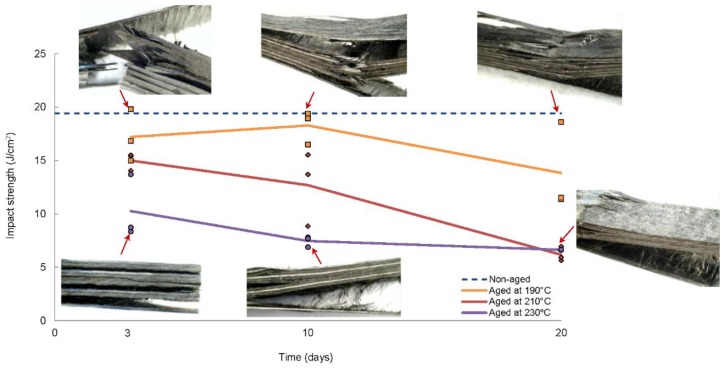
Average impact strength (*E*_c_) and failure modes of quasi-isotropic [0/90/±45]_2s_ laminates aged at different temperatures: 3, 10 and 20 days (*E*_impact_ = 32.46 J).

Finally, additional ageing treatments were performed at temperatures very close and above the glass transition temperature (*T*_g_ = 195 °C) in order to accelerate the degradation and detect a possible dependence of the mechanical properties of the CFRP laminates with the temperature and the exposure time. For that purpose, ageing treatments at 190 °C, 210 °C and 230 °C were performed on quasi-isotropic [0/90/±45]_2s_ laminates. The time of ageing considered for this study were: 3, 10 and 20 days. The values of the average maximum force and impact strength for the different ageing treatments are shown in Figure 8 and Table 6. The results revealed that both temperature and time of ageing affected significantly the impact response of CFRP laminates. On one hand, it was observed that the highest values of impact strength corresponded to the ageing treatment at 190 °C and as the ageing temperature increased, the impact strength decreased. On the other hand, comparing the energy that was absorbed at a particular temperature, it can be concluded that longer exposure time to high temperatures resulted in further degradation. A slight improvement on the average impact strength was observed after 10 days of ageing at 190 °C, however it did not seem to be associated with a post-curing process because, in that case, the improvement should have appeared from the beginning and overcame the value for the non-aged laminates. A possible explanation for this apparent increase could be the dispersion of the results as a consequence of the manufacturing technique (hand lay-up), that frequently causes the presence of discontinuities in the laminates. The same explanation would explain the higher values of absorbed energy of the laminates aged at 230° than those at 210 °C after 20 days of ageing.

As shown in Figure 8, one of the most remarkable effects of ageing was the change in the failure mode. In specimens aged at 190 °C (close to *T*_g_) for short periods of time, the main failure mode were fibre breakage and delamination, the same as non-aged specimens. However, as temperature and time of ageing increased, the polymeric matrix experienced a significant degradation, decreasing the adhesion between the matrix and the fibres. Thus, failure occurred mainly through the matrix resulting in lower energetic failure mode.

**Figure 9:**
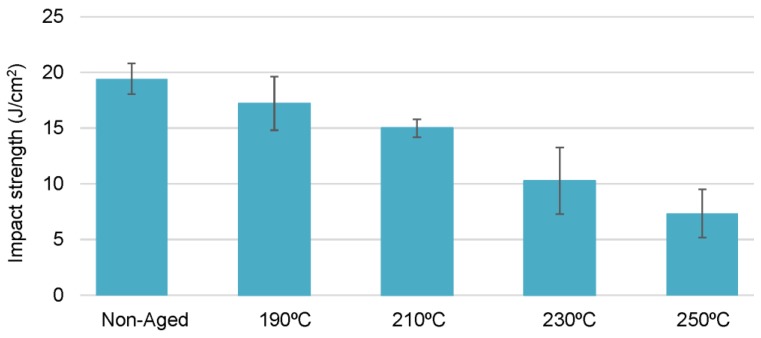
Average impact strength (*E*_c_) of quasi-isotropic [0/90/±45]_2s_ laminates aged at different temperatures for 3 days (*E*_impact_ = 32.46 J).

It is important to note that ageing treatments at temperatures very close and above the glass transition temperature caused remarkable degradation in the impact resistance of CFRP laminates, even for short periods of time (3 days), as it is shown in Figure 9. This behaviour contrasts with the observed at 150 °C as it was discussed previously.

### 3.2. Effect of Ageing on the Flexural Performance

A series of three-point bending tests were performed in thermal aged quasi-isotropic [0/90/±45]_2s_ laminates in order to determine the effect of temperature on the flexural properties of CFRP laminates. 

The effect of ageing on the flexural resistance was evaluated by means of average maximum flexural stress, flexural modulus and flexural strain (Table 7). Additionally, optical micrographs of the cross-sections of the samples were examined to gain an insight into the assessment of different failure mechanisms. Three-point bending tests were conducted at room temperature. 

**Figure 10:**
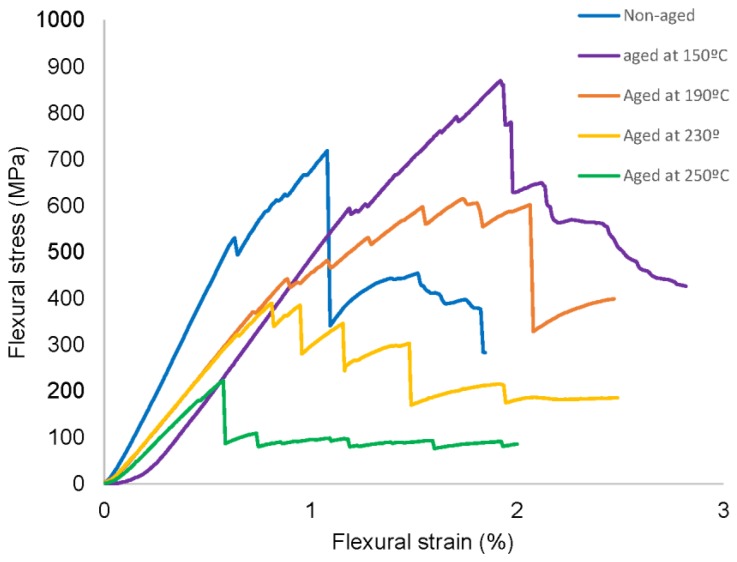
Flexural stress-strain curves of quasi-isotropic [0/90/±45]_2s_ laminates aged at different temperatures for 10 days.

Figure 10 shows flexural stress-strain curves for the different [0/90/±45]_2s_ quasi-isotropic laminates aged at different temperatures for 10 days. Temperatures below and above the glass transition temperature were considered. The curve corresponding to non-aged quasi-isotropic laminate was also included for comparative purposes. The maximum flexural stress gradually decreased as temperature of ageing increased, as it was expected, except for specimens aged at 150 °C, which depicted the highest value of flexural stress, 15% higher than non-aged specimens. Thus, the post-curing effect was also observed on flexural samples, similarly to impact samples. The decrease of the flexural properties of aged CFRP laminates could be mainly attributed to changes of the molecular configuration accompanied by a hydrothermal deterioration. The maximum flexural stress was reduced significantly, with a decrease of 73% compared to its original value, after 10 days at 250 °C. The reason was the degradation of the polymer matrix as a consequence of the thermo-oxidative environment. In addition, it should be noticed that in the case of the flexural stiffness, it did not exhibit the post-curing effect as observed in the maximum flexural stress at 150 °C. On the contrary, the flexural modulus of aged specimens was considerably lower than the non-aged ones but no significant variations with the ageing temperature were observed. The maximum decrease of flexural modulus was around 57% for specimens aged at 250 °C for 10 days. Furthermore, it was observed an increase of flexural strain with temperature until reaching the maximum value at temperatures very close to the *T*_g_. The reason was the loss of flexural stiffness with the increase of the thermal ageing temperatures. After the glass transition temperature, the flexural strain decreased, due to an embrittlement of the epoxy resin. These results were in agreement with previously works [7,8,18,19].

**Figure 11:**
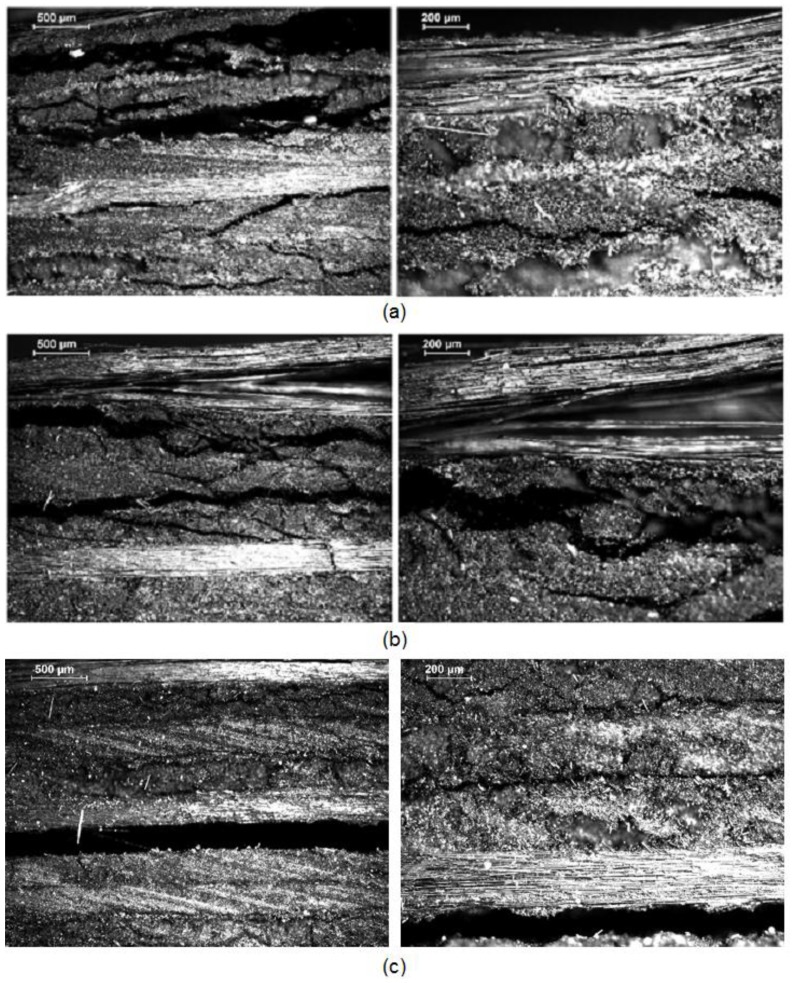
Cross sectional optical micrographs of the failure areas of flexural quasi-isotropic [0/90/±45]_2s_ samples. (**a**) Non-Aged; (**b**) aged at 190 °C for 10 days, (**c**) aged at 250 °C for 10 days.

Figure 11 shows optical micrographs of the cross-sections of the flexural failed samples (non-aged, aged at 190 °C and aged at 250 °C) in order to gain an insight into the assessment of different failure as a consequence of the thermal ageing. Non-aged specimens showed damage initiation at the compressive side of the specimen and delamination and fibre/matrix interfacial debonding were observed in the upper layers, close to the applied load, according to typical flexural failure of quasi-isotropic laminates [39]. The decrease in the flexural strength of aged specimens might be attributed to the development of microcracks due to the oxidation and degradation of the matrix and the fibre/matrix interface. Micrographs verified such behaviour, where crack density and delamination significantly increased as ageing temperature increased. Further thermal degradation was observed in specimens aged at 250 °C because of an increase of matrix cracking and delamination.

**Figure 12:**
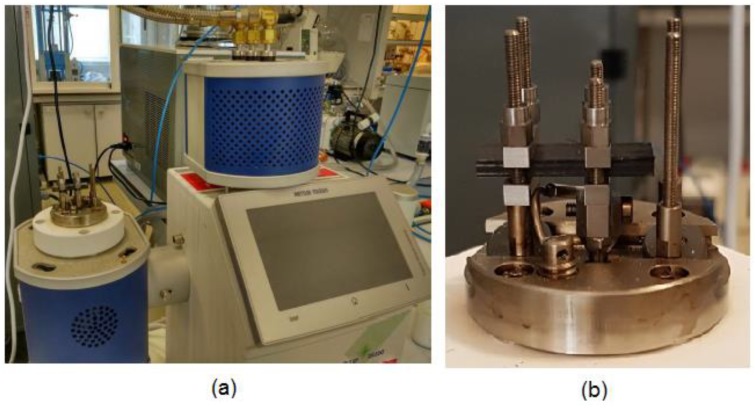
(**a**) DMA Metller Toledo equipment and (**b**) details of the cantilever beam configuration setup according to ASTM D7028 standard.

Finally, Dynamic Mechanical Analysis (DMA) technique according to ASTM D7028 [48] standard with a cantilever beam configuration have been carried out in a Metller Toledo equipment (Figure 12) in order to obtain the glass transition temperatures (*T*_g_) of the flexural samples with different aging treatments. ASTM D7028 recommends the use of either a cantilever beam or three-point bending flexural loading for *T*_g_ determination of polymer matrix composites. DMA method applied an oscillating force to the samples and measured the resulting displacement as the test temperature was slowly increased. From the measured force and displacement, the specimen stiffness was determined and used to identify the glass transition temperature (*T*_g_). The results of the different glass transition temperatures for the flexural samples with different aging treatments are depicted in the Table 8. Three samples of each type were analysed and the average value was used as the final result.

The results showed a slight drop of *T*_g_ for aged samples below the initial *T*_g_ (150 °C and 190 °C). However, the reduction of *T*_g_ was more significant for aged samples over the initial *T*_g_ (230 °C and 250 °C). These downward trends are in agreement with those depicted in the impact and flexural performances of aged CFRP samples. The results are in accordance with previous works [7,8,18,20].

## 4. Conclusions

The purpose of this study was to investigate the effect of thermal ageing on carbon fibre-reinforced epoxy composites exposed at different temperatures below and above the *T*_g_ and different periods of time. Instrumented Charpy and three-point bending tests were performed in order to determine the impact of thermal ageing on the mechanical performance. The main conclusions of this work are shown below:

- Samples aged at 150 °C (*T* < *T*_g_): a consolidation phase was observed that caused a post-curing effect. High temperature enhanced the crosslink density in the material and consequently, there was an increase in both impact and bending strength but a decrease in flexural stiffness.
- Samples aged at 190 °C (*T* ≈ *T*_g_): the effect of temperature and time of ageing caused a progressive decrease of mechanical properties with an increase in delaminations and microcracks but no sign of consolidation stage were observed.
- Samples aged at 230–250 °C (*T* > *T*_g_): further thermal degradation of the matrix occurred for temperatures that exceeded the glass transition temperature which implied the decomposition of the fibre-matrix interface and the reduction of the impact resistance and mechanical performance. The results depicted a significant change on the impact and flexural performance of aged CFRP laminates compared to the rest of the samples.

It can be concluded that both temperature and time of ageing were critical parameters to be consider on the design of structural polymer composite components in order to ensure the long-term performance of these structures. 

## Figures and Tables

**Table 1:** Stiffness and Strength properties of the M21E/IMA carbon fibre/epoxy system.

**E_11T_ (GPa)**	**E_22T_ (GPa)**	**G_12_ (GPa)**	$\sigma_{11T}$ **(MPa)**	$\sigma_{22T}$ **(MPa)**	$\tau_{12}$ **(MPa)**
178	11.8	5.2	3050	56	95
**E_11C_** **(GPa)**	**E_22C_** **(GPa)**	$\vartheta_{12}$	$\sigma_{11C}$ **(MPa)**	$\sigma_{22C}$ **(MPa)**	
146	7.4	0.39	1500	200	

**Table 2:** Thermal ageing treatments for the carbon fibre-reinforced epoxy composites.

Thermal Ageing Impact Samples		Thermal Ageing Flexural Samples	
Temperature (°C)	Time (days)	Temperature (°C)	Time (days)
150	28, 35, 42	150	10
190	3, 10, 20	190	10
210	3, 10, 20	230	10
230	3, 10, 20	250	10

**Table 3:** Impact characteristic of the drop-weight test.

Drop Height	Impact Velocity	Impact Mass	Impact Energy (*E*_impact_)
736 mm	3.8 mm/s	4.5 kg	32.46 J

**Table 4:** Maximum force and maximum impact strength results of non-aged laminates subjected to an impact energy of 32.46 J.

Impact Samples	[0/90/±45]_2s_		[0/90]_4s_	
	*F*_max_ (N)	*E*_c_ (J/cm^2^)	*F*_max_ (N)	*E*_c_ (J/cm^2^)
P1	3726	18.51	5446	25.25
P2	3125	18.51	5469	24.74
P3	3809	21.43	5470	28.71
P4	3875	19.25	4688	11.98
P5	3568	19.01	5155	18.04
P6	3586	21.00	5500	28.00

**Table 5:** Average maximum force and impact strength results of quasi-isotropic [0/90/±45]_2s_ laminates subjected to an impact energy of 32.46 J after an ageing treatment at 150 °C (*T* < *T*_g_). Standard deviation is included.

[0/90/±45]_2s_ (*T* = 150 °C)		
Time (days)	*F*_max_ (N/cm^2^)	*E*_c_ (J/cm^2^)
28	3509 ± 71	21.2 ± 1.1
35	3667 ± 226	22.1 ± 1.6
42	3527 ± 144	20.5 ± 1.3

**Table 6:** Average maximum force and impact strength results of quasi-isotropic [0/90/±45]_2s_ laminates subjected to an impact energy of 32.46 J after different ageing treatments (*T* ≈ *T*_g_ and *T* > *T*_g_). Standard deviation is included.

[0/90/±45]_2s_			
Temperature (°C)	Time (days)	*F*_max_ (N/cm^2^)	*E*_c_ (J/cm^2^)
190	3	3042 ± 140	17.2 ± 1.9
	10	2896 ± 80	18.3 ± 1.3
	20	2999 ± 166	13.8 ± 3.4
210	3	2693 ± 299	15.0 ± 0.7
	10	2214 ± 254	12.7 ± 2.8
	20	1862 ± 48	6.2 ± 0.6
230	3	2150 ± 135	10.3 ± 2.4
	10	2224 ± 41	7.5 ± 0.4
	20	2319 ± 129	6.6± 0.1

**Table 7:** Average values of maximum flexural stress, flexural modulus of elasticity and maximum flexural strain of quasi-isotropic laminates aged at different temperatures for 10 days. Standard deviation is included.

Temperatures	σ (MPa)	*E* (GPa)	ε (%)
Non–Aged	724 ± 52	81.2 ± 4.9	1.46 ± 0.11
Aged at 150 °C	835 ± 58	54.8 ± 4.7	2.27 ± 0.21
Aged at 190 °C	608 ± 74	49.9 ± 7.1	2.41 ± 0.01
Aged at 230 °C	417 ± 24	48.1 ± 4.4	1.43 ± 0.13
Aged at 250 °C	193 ± 26	45.9 ± 10.1	0.91 ± 0.18

**Table 8:** Average values of glass transition temperature (*T*_g_) of quasi-isotropic laminates aged at different temperatures for 10 days. Standard deviation is included.

Aging Treatment	*T*_g_ (°C)
Non–Aged	194.8 ± 0.3
Aged at 150 °C	192.6 ± 0.2
Aged at 190 °C	189.2 ± 0.4
Aged at 230 °C	175.3 ± 0.5
Aged at 250 °C	171.3 ± 0.2

## References

[1] Shi Y., Swait T., Soutis C. (2012). Modelling damage evolution in composite laminates subjected to low velocity impact. Compos. Struct..

[2] Selver E., Potluri P., Hogg P., Soutis C. (2016). Impact damage tolerance of thermoset composites reinforced with hybrid commingled yarns. Compos. Part B Eng..

[3] Shi Y., Soutis C. (2016). Modelling transverse matrix cracking and splitting of cross-ply composite laminates under four point bending. Theor. Appl. Fract. Mech..

[4] Farooq U., Myler P. (2015). Prediction of load threshold of fibre-reinforced laminated composite panels subjected to low velocity drop-weight impact using efficient data filtering techniques. Results Phys..

[5] Soutis C. (2005). Fibre reinforced composites in aircraft construction. Prog. Aerosp. Sci..

[6] Li Z., Haigh A., Soutis C., Gibson A., Sloan R., Karimian N. (2016). Detection and evaluation of damage in aircraft composites using electromagnetically coupled inductors. Compos. Struct..

[7] Barbosa A.P.C., Fulco A.P.P., Guerra E.S.S., Arakaki F.K., Tosatto M., Costa M.C.B., Melo J.D.D. (2017). Accelerated aging effects on carbon fiber/epoxy composites. Compos. Part B Eng..

[8] Ernault E., Richaud E., Fayolle B. (2017). Origin of epoxies embrittlement during oxidative ageing. Polym. Test..

[9] Marouani S., Curtil L., Hamelin P. (2012). Ageing of carbon/epoxy and carbon/vinylester composites used in the reinforcement and/or the repair of civil engineering structures. Compos. Part B Eng..

[10] Park S.Y., Choi W.J., Choi C.H., Choi H.S. (2019). An experimental study into aging unidirectional carbon fiber epoxy composite under thermal cycling and moisture absorption. Compos. Struct..

[11] Pecora M., Pannier Y., Lafarie-Frenot M.-C., Gigliotti M., Guigon C. (2016). Effect of thermo-oxidation on the failure properties of an epoxy resin. Polym. Test..

[12] Decelle J., Huet N., Bellenger V. (2003). Oxidation induced shrinkage for thermally aged epoxy networks. Polym. Degrad. Stab..

[13] Daghia F., Zhang F., Cluzel C., Ladevèze P. (2015). Thermo-mechano-oxidative behavior at the ply’s scale: The effect of oxidation on transverse cracking in carbon–epoxy composites. Compos. Struct..

[14] Bellini C., Parodo G., Polini W., Sorrentino L. (2018). Experimental investigation of hydrothermal ageing on single lap bonded CFRP joints. Procedia Struct. Integr..

[15] Wang Z., Xian G., Zhao X.-L. (2018). Effects of hydrothermal aging on carbon fibre/epoxy composites with different interfacial bonding strength. Constr. Build. Mater..

[16] Park J.-M., Shin P.-S., Kim J.-H., Baek Y.-M., Park H.-S., DeVries L.K., Naguib H.E. (2018). Evaluation of thermally-aged carbon fiber/epoxy composites using acoustic emission, electrical resistance, contact angle and thermogram. Behavior and Mechanics of Multifunctional Materials and Composites XII.

[17] Barile C., Casavola C., Pappalettere C., Tursi F. (2010). RFI composite materials behaviour. Struct. Integr. Life.

[18] Smirnova V.E., Popova E.N., Svetlichnyi V.M., Myagkova L.A., Orekhov A.N., Yudin V.E., Muzafarov A.M., Tatarinova E.A. (2011). Effect of thermal aging on the mechanical characteristics of a composite of a polyimide with an organosilicon resin. Russ. J. Appl. Chem..

[19] Belaid S., Chabira S.F., Balland P., Sebaa M., Belhouideg S. (2015). Thermal aging effect on the mechanical properties of polyester fiberglass composites. J. Mater. Environ. Sci..

[20] Merino-Pérez J.L., Ayvar-Soberanis S., Merson E., Hodzic A. The influence of heat during short ageing periods on the mechanical properties of CFRP composites. Proceedings of the 16th European Conference on Composite Materials.

[21] Zhang D., He M., He W., Zhou Y., Qin S., Yu J. (2017). Influence of Thermo-Oxidative Ageing on the Thermal and Dynamical Mechanical Properties of Long Glass Fibre-Reinforced Poly(Butylene Terephthalate) Composites Filled with DOPO. Materials.

[22] Abenojar J., Pantoja M., Martínez M.A., del Real J.C. (2015). Aging by moisture and/or temperature of epoxy/SiC composites: Thermal and mechanical properties. J. Compos. Mater..

[23] Lévêque D., Schieffer A., Mavel A., Maire J.-F. (2005). Analysis of how thermal aging affects the long-term mechanical behavior and strength of polymer–matrix composites. Compos. Sci. Technol..

[24] Tcharkhtchi A., Farzaneh S., Abdallah-Elhirtsi S., Esmaeillou B., Nony F., Baron A. (2014). Thermal Aging Effect on Mechanical Properties of Polyurethane. Int. J. Polym. Anal. Charact..

[25] Guo J., Wang M., Li L., Wang J., He W., Chen X. (2018). Effects of thermal-oxidative aging on the flammability, thermal degradation kinetics and mechanical properties of DBDPE flame retardant long glass fiber reinforced polypropylene composites. Polym. Compos..

[26] Haque M.H., Upadhyaya P., Roy S., Ware T., Voit W., Lu H. (2014). The changes in flexural properties and microstructures of carbon fiber bismaleimide composite after exposure to a high temperature. Compos. Struct..

[27] Odegard G.M., Bandyopadhyay A. (2011). Physical aging of epoxy polymers and their composites. J. Polym. Sci. Part B Polym. Phys..

[28] Hu H.W. (2007). Physical Aging in Long Term Creep of Polymeric Composite Laminates. J. Mech..

[29] Shi X., Fernando B.M.D., Croll S.G. (2008). Concurrent physical aging and degradation of crosslinked coating systems in accelerated weathering. J. Coat. Technol. Res..

[30] Sell C.G., McKenna G.B. (1992). Influence of physical ageing on the yield response of model DGEBA/poly(propylene oxide) epoxy glasses. Polymer.

[31] Caminero M.A., Garcia-Moreno I., Rodriguez G.P. (2017). Damage resistance of carbon fibre reinforcecd epoxy laminates subjected to low velocity impact: Effect of laminate thickness and ply-stacking sequence. Polym. Test..

[32] Zhang D., Sun Y., Chen L., Pan N. (2013). A comparative study on low-velocity impact response of fabric composite laminates. Mater. Des..

[33] Agrawal S., Singh K.K., Sarkar P.K. (2014). Impact damage on fibre-reinforced polymer matrix—A review. J. Compos. Mater..

[34] Akay M., Spratt G.R., Meenan B. (2003). The effects of long-term exposure to high temperatures on the ILSS and impact performance of carbon fibre reinforced bismaleimide. Compos. Sci. Technol..

[35] Gigliotti M., Pannier Y., Minervino M., Lafarie-Frenot M.C., Corigliano P. (2013). The effect of a thermo-oxidative environment on the behaviour of multistable [0/90] unsymmetric composite plates. Compos. Struct..

[36] Haridas A., Song C., Chan K., Murukeshan V.M. (2017). Nondestructive characterization of thermal damages and its interactions in carbon fibre composite panels. Fatigue Fract. Eng. Mater. Struct..

[37] Im K.-H., Cha C.-S., Kim S.-K., Yang I.-Y. (2001). Effects of temperature on impact damages in CFRP composite laminates. Compos. Part B Eng..

[38] Caminero M.A., Rodríguez G.P., Muñoz V. (2016). Effect of stacking sequence on Charpy impact and flexural damage behavior of composite laminates. Compos. Struct..

[39] Meng M., Le H.R., Rizvi M.J., Grove S.M. (2015). The effects of unequal compressive/tensile moduli of composites. Compos. Struct..

[40] Resources H. Prepeg Data Sheets. Hexply M21E Epoxy Matrix. Product data. www.hexcel.com.

[41] (2010). D6110-18 ASTM Standard Test Method for Determining the Charpy Impact Resistance of Notched Specimens of Plastics.

[42] (2010). ISO 179-1—Plastics—Determination of Charpy Impact Properties—Part 1: Non-Instrumented Impact Test.

[43] (2007). D7264/D7264M ASTM Standard Test Method for Flexural Properties of Polymer Matrix Composite Materials.

[44] Mujika F. (2007). On the effect of shear and local deformation in three-point bending tests. Polym. Test..

[45] Varna J., Joffe R., Akshantala N.V., Talreja R. (1999). Damage in composite laminates with off-axis plies. Compos. Sci. Technol..

[46] Ahmad F., Hong J.-W., Choi H.S., Park S.-J., Park M.K. (2015). The effects of stacking sequence on the penetration-resistant behaviors of T800 carbon fiber composite plates under low-velocity impact loading. Carbon Lett..

[47] Mlyniec A., Korta J., Kudelski R., Uhl T. (2014). The influence of the laminate thickness, stacking sequence and thermal aging on the static and dynamic behavior of carbon/epoxy composites. Compos. Struct..

[48] (2015). ASTM D7028-07 Standard Test Method for Glass Transition Temperature (DMA Tg) of Polymer Matrix Composites by Dynamic Mechanical Analysis (DMA).

